# China’s Provincial Eco-Efficiency and Its Driving Factors—Based on Network DEA and PLS-SEM Method

**DOI:** 10.3390/ijerph17228702

**Published:** 2020-11-23

**Authors:** Zhijun Li, Yigang Wei, Yan Li, Zhicheng Wang, Jinming Zhang

**Affiliations:** 1 School of Economics and Management, Xidian University, Xi’an 710126, China; zjli@xidian.edu.cn; 2 School of Economics and Management, Beihang University, Beijing 100191, China; 10005@buaa.edu.cn; 3 Beijing Key Laboratory of Emergency Support Simulation Technologies for City Operations, Beijing 100191, China; 4 Business School, Shandong University, Weihai 264209, China; liyan5@sdu.edu.cn (Y.L.); 201916299@mail.sdu.edu.cn (Z.W.); 5 School of Political Studies, Nanjing Agricultural University, Nanjing 210095, China

**Keywords:** eco-efficiency, network DEA, DPSIR model, PLS-SEM, environmental treatment

## Abstract

This study aims to estimate the eco-efficiencies of China at provincial levels. The eco-efficiencies of production and treatment stages are disentangled by the network data envelopment analysis (DEA) method. The key driving factors are identified by the integrative use of driving force-pressure-state-impact-response frame model (DPSIR) model and partial least squares structural equation modeling (PLS-SEM) method. This study provides several important findings. In general, the eco-efficiencies of most regions in China are inefficient and show significant regional differences. All DPSIR factors have significant and strong impacts on the eco-efficiency of the treatment stage. The eco-efficiency of the production stage evidently outweighs the eco-efficiency in economically well-developed regions. The originality of this study lies in three aspects. First, using two-stage network DEA, this study dissects the overall eco-efficiency into production efficiency and treatment efficiency. Empirical results provide insights into the root cause of the low efficiency of each province (municipality). Second, on the basis of the DPSIR model, an expanded pool of driving factors is investigated. Third, using the PLS-SEM method to analyze eco-efficiency is more reliable and effective than applying other traditional regression models.

## 1. Introduction

China’s economy has achieved rapid progress since the reform and opening up policy initiated in 1978. However, given the unprecedented economic achievements, rapid development in the past three decades has also exerted immeasurable influence on the ecological environment. Eco-efficiency means “do more with less”, which refers to the marginal impact of economic activities on the natural environment. The WBCSD’s (World Business Council for Sustainable Development) definition is generally accepted—limiting the consequent effect on the environment to a minimum level that is consistent with the Earth’s potential capacity over its life cycle. In recent years, the measurement of eco-efficiency has become a heated topic, and abundant relevant studies have been conducted on different scales. Moreover, many scholars have investigated the various influencing factors of eco-efficiency.

Most scholars generally regard resource consumption and environmental pollution as input indicators. In order to test relationships among the economic activities, urbanization and carbon emissions, different frameworks are developed to evaluate the eco-efficiency of different areas, industries or companies [1,2,3]. The empirical results, estimated through application of the system generalized method of moment (GMM), shows that energy consumption, economic growth and urbanization all significantly increase carbon emissions, thereby adding to serious environmental challenges in the twelve developing East Asian and Pacific countries for the period 1990–2014 [4]. Meanwhile, some studies confirmed the positive impact of GDP and population on CO_2_ emissions by a hierarchical regression model in the 50 largest world economies over the years 1990–2015 [5]. Many researches are conducted to measure and predict the carbon emissions and economic growth forces in China [6,7]. Han [8] argues that the urban employment rate has the greatest impact on carbon emission intensity, the per capita urban employment energy consumption has the least impact on carbon emission intensity and the degree of other factors in proper order is: urbanization rate, population intensity of GDP production and carbon emission density. The decomposition of total CO_2_ emissions from energy quantity and composition indicates that CO_2_ emissions will not peak in the business-as-usual scenario. CO_2_ emissions will peak at 10.69 gigatonnes (Gt) in 2030 in the planned energy structure scenario [9]. Some researchers focus on the impact of land use to carbon emission [10,11]. It was estimated that the average annual increase of carbon storage by urban forest could offset 3.9% of the average annual increase in urban carbon emissions under China’s rapid urban expansion and greening [12].

However, eco-efficiency measurements should focus not only on total factor input, but also process analysis [12]. Bostian [13] demonstrated how these developments in modeling pollution-generating technologies can be incorporated into a network model framework, with material balance conditions, black box technologies and detailed processes within the black box considered. In the terms of eco-efficiency measurement, the strand of literature on efficiency measures based on the perspective of non-parametric methodology has gained growing popularity [14]. Some studies use the data envelopment analysis (DEA) method to measure the eco-efficiency of the forest sector [15] and agricultural eco-efficiency in Italian regions [16]. In recent years, the combined use of DEA and other method has been widely adopted in assessment of eco-efficiency. Economic input-output life cycle assessment (EIO-LCA) and data envelopment analysis (DEA) are combined to assess the environmental impacts and eco-efficiency of China’s 26 economic sectors [17]. Rebolledo [18] compared the pros and cons of two methods—the integration of life cycle assessment (LCA) and DEA and the combined use of carbon footprint (CF) and DEA.

Most studies use the traditional DEA method to evaluate the ecological efficiency, and treat the input-output process as a “black box”, that is, they only focus on the input and output factors, but rarely further decompose the internal relationships between these factors. Network DEA can open the “black box” by dividing the actual input-output process into production stage and environmental governance stage [19]. By splitting the production process, network DEA can better identify the root cause of the low efficiency of each decision-making unit (DMU) than the traditional DEA [20]. The two-stage network DEA can be used in the evaluation of decision-making units (DMUs), which are composed of two or more stages of subprocesses. Therefore, the underlying logic is closer to the organizing structure in reality. Moreover, network DEA is widely applicable to environmental studies. Every input of a subprocess could be the intermediate product of the previous subprocess. It could also be an external input variable. Every output of a subprocess serves not only as an intermediate product of other inputs of the subprocess but also as an end product [21]. When examining the further relationship among the regional eco-efficiency of the driving forces in different stages, partial least squares structural equation modeling (PLS-SEM) can select all protected features from the observed data on account of fewer restrictions on the assumption of the normal distribution [22].

Many literatures have studied the influencing factors of ecological efficiency. The DPSIR framework provides an analytical framework for systematically measuring the influencing factors of ecological efficiency, namely, driving forces, pressure, state, impact and response [23]. The DPSIR analysis framework integrates the causal relationship between human activities and their environmental and socio-economic consequences into one framework, and considers human activities as an integral part of the ecosystem [24], so it is widely used in the analysis of environmental problems [25]. However, these five factors cannot be directly measured. The SEM model is a common method to solve this problem; that is, setting observation variables for latent variables that are difficult to be measured directly, and using the relationships between these observed variables that can be used for statistical analysis to study the relationships between latent variables [26]. The estimation of model parameters also needs to choose an appropriate method. In this paper, the PLS method is used to estimate the SEM model, which is mainly because the PLS-SEM model has the advantages of handling the measurement deviation of variables, no requirements on data distribution and being effective in the case of small samples [27].

The purpose of this paper is to estimate the eco-efficiency of thirty provincial regions in China, and to identify the driving factors of eco-efficiency. The contribution of this article includes the following: (1) eco-efficiency is estimated and decomposed into two stages of production by means of a two-stage DEA model. Empirical results shed light on the root cause of low efficiency in China at provincial level. (2) To investigate the driving factors, extant literature mainly focuses on a limited number of assumed variables. Based on the theoretical underpinnings of DPSIR, this research covers an expanded pool of potential variables. (3) In terms of research method, this study uses the PLS-SEM approach to examine the driving factors of eco-efficiency in different stages across 30 administrative regions at the provincial level. This method provides more robust outcomes than traditional regression models.

The rest of the paper is organized as follows. The second section introduces the research methods and data. The third and fourth sections discuss the evaluation results of ecological efficiency and the influencing factors. The fifth section concludes the study.

## 2. Research Method and Data

### 2.1. Two-Stage Network DEA

DEA is a mathematical programming model which evaluates the comparative efficiency of DMUs, calculating a relative ratio of weighted outputs to weighted inputs for each DMU and explores the best-performing frontiers over the sample data [28]. Initially DEA was implemented in the radial distance [29]. Non-radial measures were developed to take desirable properties into consideration [30,31,32]. Since then, Cooper and Pastor [33], Briec [34], Cooper et al. [35] and Pastor et al. [36] developed the DEA framework. The method is applied to estimate the efficiency or effectiveness of a system based on various input and output indicators [37]. The DEA method has some unique advantages in calculating the efficiency of the DMU [38]. The latest network DEA model considers and connects the indirect input and outputs during a complete economic production process and network. Specifically, two stage DEA is a commonly used model of network DEA. By decomposing the input-output process into production process and environmental governance process, the “black box” of the relationship between elements is opened [39].

First seen in Liang et al. [40], the two-stage DEA is a model that originated from cooperative game and non-cooperative game. The two parts refer to the stages of production and pollutant treatment. In this study, economic activities are divided into two stages, namely, production stage and pollutant treatment stage. Then, the two-stage DEA model is used to calculate and evaluate the comprehensive efficiency. To use this framework, the definition of input, intermediate variable and output must be clarified first. The jth decision making unit is represented by ${\mathrm{DMU}}_{j}\left(j\;\epsilon {\;N}^{*}\cap^{\;}\left(1,J\right)\right)$, and each DMU refers to a provincial-level administrative region in China. We assume that ${\mathrm{DMU}}_{j}$ has initial inputs $X_{\mathrm{nj}}\left(n\;\epsilon {\;N}^{*}\cap^{\;}\left(1,N\right)\right)$ and final outputs $Y_{\mathrm{rj}}\left(r\;\epsilon {\;N}^{*}\cap^{\;}\left(1,R\right)\right)$ with intermediate desirable outputs $V_{\mathrm{mi}}\left(m\;\epsilon {\;N}^{*}\cap^{\;}\left(1,M\right)\right)$ and intermediate undesirable outputs $W_{\mathrm{kj}}\left(k\;\epsilon {\;N}^{*}\cap^{\;}\left(1,K\right)\right)$. For phase two, the inputs are set to undesirable outputs W, along with a new external input $Z_{\mathrm{tj}}\left(t\;\epsilon {\;N}^{*}\cap^{\;}\left(1,T\right)\right)$.

**Figure 1:**
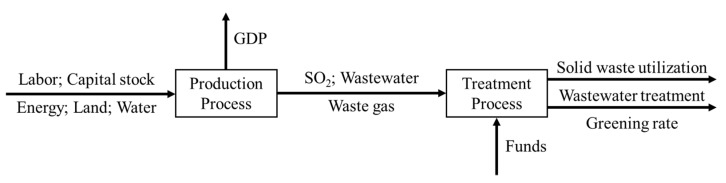
Conceptual framework of two stages of eco-efficiency. (Source: Authors compiled).

Figure 1 depicts the internal relationship of the model, involving input, intermediate and output variables. In the first stage (production process), input variables include labor, capital stock, energy, land and water and the expected output is GDP. At the same time, in the cycle of production, other pollution issues such as waste gas, wastewater and SO_2_, which are undesirable output variables, emerged. In the second stage (treatment process), input variables are divided into two parts. The first part refers to the serious pollutants that need to be noted and treated in the first stage, including waste gas, wastewater and SO_2_. The second part derives from the government’s enormous funding in environmental protection every year, and as new input, it enters the second stage as well. The outputs of the second stage include solid waste utilization, wastewater treatment and greening rate. Among them, the greening rate refers to the ratio of the greenbelt area within the scope of construction land to the construction land area. In addition to pollution control, the government’s investment, represented by Z, also promotes the advancement of the process. In this stage, the final output is energy consumption, followed by the reduction of pollutant emissions. The next segment will provide more definitions and the indicator analysis. The DEA model described in this study can be expressed by the following linear optimization model.

(1) $$\begin{array}{c} \theta_{0}^{s1}=\max\frac{1}{2}\left(\frac{1}{N}\overset{N}{\underset{n=1}{\sum }}\theta_{n}+\frac{1}{K}\overset{K}{\underset{k=1}{\sum }}\alpha_{k}\right)\\ s.t.\overset{J}{\underset{j=1}{\sum }}\lambda_{j}{Χ}_{\mathrm{nj}}\leq \theta_{n}{Χ}_{\mathrm{nj}0},n\;\epsilon {\;N}^{*}\cap^{\;}\left(1,N\right),\\ \overset{J}{\underset{j=1}{\sum }}\lambda_{j}V_{\mathrm{mj}}\geq V_{\mathrm{mj}0},m\;\epsilon {\;N}^{*}\cap^{\;}\left(1,M\right),\\ \overset{J}{\underset{j=1}{\sum }}\lambda_{j}W_{\mathrm{kj}}=\alpha_{k}W_{\mathrm{kj}0},k\;\epsilon {\;N}^{*}\cap^{\;}\left(1,K\right),\\ \overset{J}{\underset{j=1}{\sum }}\lambda_{j}=1,\lambda_{j}\geq 0,\forall j\epsilon {\;N}^{*}\cap^{\;}\left(1,J\right) \end{array}$$

In model (1), $\lambda ={\left(\lambda_{1},\lambda_{2},\ldots ,\lambda_{j}\right)}^{T}$ refers to intensive variables connected by a convex combination to the input and output of each DMU. The constraint of the sensitivity variables $\lambda_{j}$ to a limit of 1 returns to scale. Furthermore, the implementation of x and v inequality constraints models the high input disposability and desirable outputs. Undesirable outputs are slightly less disposable, and the equity constraint is assured. Apart from the limitations, two worthwhile observations are made. Next, the control factors $\theta_{n}$ and $\alpha_{k}$ are used to decrease the inputs and unnecessary outputs.

Notably, these reduction factors are fixed for each DMU. The reduction of unity is unified among the regions. Then, the objective function is divided into two parts. The weight of each part is 0.5, which refers to the pollution output effect $\alpha_{k}$ and the energy input effect $\theta_{n}$, respectively. Given the conditions of phase one, we can characterise the unified energy and environmental efficiency in the production process as $\theta_{0}^{\mathrm{slm}}$, which is called the production process efficiency (proeff). If proeff = 1, then the corresponding DMU will be considered inefficient. Thus, the consumption and emissions could be reduced. When the efficiency value of multiple DMUs is equal to 1, that is, when there are multiple effective DMUs, we adopt the super efficiency DEA model [37] to sort them. The specific operation of the super efficiency DEA model is: when evaluating the efficiency of a decision-making unit, it is excluded firstly. In the evaluation, for the invalid DMU, its production frontier is unchanged, so its final efficiency value is the same as that measured by the traditional DEA model; for the effective DMU, on the premise of its efficiency value unchanged, the input increases proportionally, and the proportion of input increase is recorded as the super efficiency evaluation value. As the production front moves backward, the measured efficiency value should be greater than or equal to 1 [41]. Stage two introduces the DEA model as follows.

(2) $$\begin{array}{c} \theta_{0}^{S^{2}}=\max\frac{1}{T}\overset{T}{\underset{t=1}{\sum }}\beta_{t}\\ s.t.\overset{J}{\underset{j=1}{\sum }}\lambda_{j}Z_{\mathrm{tj}}\leq \beta_{t}Z_{\mathrm{tj}0},t\;\epsilon {\;N}^{*}\cap^{\;}\left(1,T\right),\\ \overset{J}{\underset{j=1}{\sum }}\lambda_{j}\Upsilon_{\mathrm{rj}}\geq \Upsilon_{\mathrm{rj}0},r\;\epsilon {\;N}^{*}\cap^{\;}\left(1,R\right),\\ \overset{J}{\underset{j=1}{\sum }}\lambda_{j}W_{\mathrm{kj}}=W_{\mathrm{kj}0},k\;\epsilon {\;N}^{*}\cap^{\;}\left(1,K\right),\\ \overset{J}{\underset{j=1}{\sum }}\lambda_{j}=1,\lambda_{j}\geq 0,\forall j\;\epsilon {\;N}^{*}\cap^{\;}\left(1,J\right) \end{array}$$

In the expression, λ refers to the same variable as that in model (1). For each DMU to be evaluated, its undesirable output w, as well as the linear combination of the undesirable output of all j DMUs, are the same. We assume that the pollutants created in the first stage would all enter the second stage without reduction. Variable βt represents the change of efficiency after pollution treatment with government funding.

$\theta_{0}^{s2}$ denotes the energy-saving treatment efficiency. In addition, stage two emission reduction is called treatment efficiency (TREATEFF). If TREATEFF = 1, then the DMU to be calculated is considered effective in stage two. However, the corresponding DMU is inefficient if TREATEFF < 1, thus making the treatment of emissions more effective. For a comprehensive view of the two stages, model (3) provides the overall efficiency of the two stages. According to Liang et al. [40], the reduction of intermediate measures to acquire optimal efficiency scores is collaboratively determined by the two stages. These stages simulate real-life scenarios, such as the practice of pricing goods together by the manufacturer and the retailer, to maximize the profit. In a cooperative game, both stages’ efficiency scores are maximized in the meantime. The model below calculates the scores:

(3) $$\begin{array}{c} \theta_{0}^{\mathrm{all}}=\max\frac{1}{2}\left(\frac{1}{N+K}\left(\overset{N}{\underset{n=1}{\sum }}\theta_{n}+\overset{K}{\underset{k=1}{\sum }}\alpha_{k}\right)+\frac{1}{T+K}\left(\overset{T}{\underset{t=1}{\sum }}\beta_{t}+\overset{K}{\underset{k=1}{\sum }}\alpha_{k}\right)\right)\\ s.t.\\ \mathrm{stage}1:\overset{J}{\underset{j=1}{\sum }}\lambda_{j}{Χ}_{\mathrm{nj}}\leq \theta_{n}{Χ}_{\mathrm{nj}0},n\;\epsilon {\;N}^{*}\cap^{\;}\left(1,N\right),\\ \overset{J}{\underset{j=1}{\sum }}\lambda_{j}V_{\mathrm{mj}}\geq V_{\mathrm{mj}0},\;m\;\epsilon {\;N}^{*}\cap^{\;}\left(1,M\right),\\ \overset{J}{\underset{j=1}{\sum }}\lambda_{j}W_{\mathrm{kj}}=\alpha_{k}W_{\mathrm{kj}0},\;k\;\epsilon {\;N}^{*}\cap^{\;}\left(1,K\right),\\ \mathrm{stage}2:\overset{J}{\underset{j=1}{\sum }}\lambda_{j}Z_{\mathrm{tj}}\leq \beta_{t}Z_{\mathrm{tj}0},t\;\epsilon {\;N}^{*}\cap^{\;}\left(1,T\right),\\ \overset{J}{\underset{j=1}{\sum }}\lambda_{j}Y_{\mathrm{rj}}\geq Y_{\mathrm{rj}0},r\epsilon {\;N}^{*}\cap^{\;}\left(1,R\right),\\ \overset{J}{\underset{j=1}{\sum }}\lambda_{j}W_{\mathrm{kj}}=\alpha_{k}W_{\mathrm{kj}0},k\epsilon {\;N}^{*}\cap^{\;}\left(1,K\right)\\ \overset{J}{\underset{j=1}{\sum }}\lambda_{j}=1,\lambda_{j}\geq 0,\forall j\epsilon {\;N}^{*}\cap^{\;}\left(1,J\right) \end{array}$$

In these two stages, W is regarded as an unacceptable output and should be reduced to minimize the quality of pollution. The target function incorporates two parts, each representing the production stage and the pollutant treatment stage. $\theta_{0}^{\mathrm{all}}$ is defined as the two-stage global level of efficiency and is generally called ALLEFF. If ALLEFF = 1, then the efficiency of the DMU to be evaluated is recognized in the global two stages. Otherwise, this DMU is considered inefficient.

### 2.2. PLS-SEM Model

This work further examines the relationship between provincial regional eco-efficiency in China and its driving factors using an SEM model. Structural equation model (SEM) is a multivariate estimation method which can effectively test the relationship between indexes and latent variables, latent variables and latent variables [42]. There have been two major methods in estimating the parameters of SEM. The PLS method to SEM has been proposed as a component-based estimation procedure different from the classical covariance-based approach [43]. 

The covariance-based technique adopts maximum likelihood-based estimations and thus the difference between the estimated and sample covariance matrices is minimized. In contrast, maximization of the explained variance of the endogenous latent variable(s) is performed by the PLS-SEM which estimates partial model relationships in an interactive sequence of ordinary least squares regressions [44]. The estimation process is per se an iterative algorithm that separately solves the blocks out of the estimation model and then calculates the path coefficients in the SEM model. The latent variable scores estimated by the PLS-SEM modeling technique are the exact linear combination of their associated manifest variables. The latent variable scores are also recognized as perfect substitutes for the manifest variables. The scores capture the variance information, which sheds light on understanding the endogenous latent variable(s). The estimation model based on ordinary least squares regressions suggests that the PLS-SEM “relaxes” the presumption of the multivariate normality required for maximum likelihood-based SEM estimations [44].

This paper further examines the relationship among provincial regional eco-efficiency in China and its driving factors using a PLS-SEM model. The structural equation consists of measurement equation and structural equation. The measurement equation refers to the link between indexes and latent variables, whereas the structural equation refers to the relationship between latent variables. The measurement equation is expressed as follows:

(4) $$\begin{array}{c} X=\Lambda_{X}\xi +\delta \\ Y=\Lambda_{Y}\eta +\varepsilon \end{array}$$

In the above formula, X and Y represent the vector composed of exogenous index and endogenous index, respectively, ξ and η represent the vector composed of exogenous latent variable and endogenous latent variable, respectively, $\Lambda_{X}$ and $\Lambda_{Y}$ are corresponding parameters to be estimated, δ and ε are disturbance terms. The structural equation is expressed as follows:

(5) η=Bη+Γξ+ζ

In the above formula, B represents the relationship between endogenous latent variables. Γ indicates the influence of exogenous latent variable on endogenous latent variable, ζ is error term. In Equation (4) and Equation (5), PLS is used to estimate the measurement sample, and the convergence coefficient is obtained by many iterations. The covariance-based technique includes constraints on the size of the data sample. A small-sized sample usually causes biased estimation and identification issues. Therefore, the PLS-SEM model proposes “soft” distributional assumptions regarding the data distributions, the data size and the measurement scale. In addition, because the PLS-SEM model is based on OLS regressions, it generally shows a good estimation performance statistical power [45].

The verification of the structural model is mainly composed of internal consistency, reliability verification and convergent validity verification. Composite reliability is used to evaluate internal consistency. When composite reliability falls between 0 and 1, the higher the value, the more reliable it is (better if the value is bigger than 0.7). The convergent validity is evaluated through measuring the factor loading and average variance extracted. The interaction between latent variables and measured variables is represented by factor loading, and 0.7 is set as the cut-off value. AVE (average variance extracted) denotes the degree to which latent variables could explain variation, and the estimated value of it is higher than 0.5.

The demonstration of the structural model involves two aspects, which are path coefficient and the significance testing of the coefficient. In the first part, the path coefficient represents the variation of the external latent variables caused by the unit change of the internal latent variables. The estimated value of the coefficient reflects the degree to which external latent variables and internal latent variables correlate with each other. The second part is the significant test of the path coefficient, which examines whether external latent variables would significantly influence internal latent variables. The significance of each t was evaluated by calculating the general value.

Requirement is the laxest among the PLS-SEM criteria on model assumption, which also includes calculation scope, sample number and residual distribution [46]. This modeling method can select all protected features from the observed data [22]. Using a PLS-SEM model, this study tests the effect of critical factors on China’s eco-efficiency.

PLS-SEM’s improvements are summarized as follows. First, when using the PLS method to estimate the SEM model, there is no restriction on variable distribution requirements, while CB-SEM (Covariance-Based SEM) model requires variables to obey normal distribution [47]. Secondly, when the sample size is very small, PLS-SEM estimates have good consistency, while CB-SEM cannot obtain robust results [27]. Third, if the model lacks a reliable theoretical basis and if the direction of the relationship between variables cannot be determined, CB-SEM should not be used as a method of choice. In contrast, PLS-SEM can examine structures and relationships in complex structural models. Since the main purpose of theoretical development is to find relationships, the direction and advantages of relationships and observable measures, PLS-SEM model is more suitable for this study.

### 2.3. Data Sources

On the basis of the established eco-efficiency evaluation system, 30 provincial administrative regions and municipalities in China from 1996 to 2015 are selected as the decision-making units. Thus, data of input and output are collected. The data set comes from each province’s statistical yearbook of the corresponding year. Following the practice of Wang and Feng [19] and making appropriate expansion, this paper selects input-output indicators as shown in Table 1. When the DEA method calculates efficiency, it first obtains the weight coefficient of each output and input index according to the data itself, so as to calculate the weighted output and weighted input, and define their ratio as the efficiency value. Therefore, the unit of the indicator does not affect the setting of the weight, and the efficiency value determined by the weighted input and the weighted output is also a de-unitized value.

## 3. Result and Discussion

This study analyses the eco-efficiency of 30 provincial-level administrative regions in mainland China (except Tibet) and horizontally compares each province’s eco-efficiency. The next section presents and discusses the empirical results.

### 3.1. Analysis of Regional Eco-Efficiency of Different Provincial Administrative Regions in China

**Figure 2:**
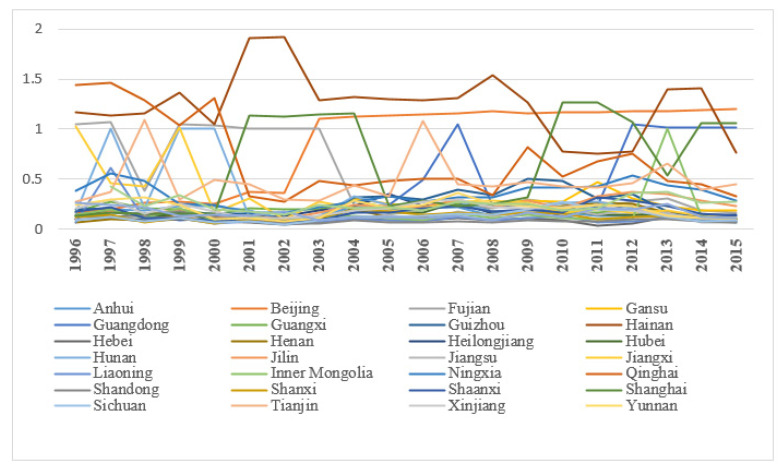
The comprehensive provincial eco-efficiency of China. (Source: Authors compiled).

In the process of numerical estimation, this study established a super-efficiency DEA model and calculated the eco-efficiency of 30 provincial administrative regions in China from 1996 to 2015 by using MaxDEA software. Table 2 is the calculation result, and the yearly change in the regional eco-efficiency of the different regions is displayed in Figure 2.

Figure 2 clearly demonstrates that the overall provincial eco-efficiency of China displays different changing trends in various stages. However, the eco-efficiency of most of the provincial administrative regions in China is not in the efficient range on the whole. As shown in Figure 2, changes in the eco-efficiency of the 30 Chinese provinces can be categorized into four patterns, namely, rising, fluctuating rising, fluctuating falling and fluctuating. Specifically, the eco-efficiency of Beijing steadily increased from 1996 to 2015. Then, eco-efficiency of Hainan, Tianjin and Shanghai showed drastic fluctuation. In addition, the fluctuation of the eco-efficiency of Qinghai, Fujian, Jiangxi and Hunan decreased, while that of Guangdong displayed an opposite trend. Therefore, fluctuations in eco-efficiency only occurred in a few provinces, and the comprehensive eco-efficiency of most cities fell between 0 and 0.5. Moreover, the volatility was minor, which shows that the eco-efficiency of most Chinese regions was in the inefficient range. China’s economy growth has been traditionally driven by the secondary industry which contributed a substantial weight to regional GDP. Secondary industry in China mainly consists of energy- and pollution-intensive enterprises. Therefore, the ecological performance of most provinces is at a low level [48]. Thus, China should promote a balanced development between economic growth and ecological environment protection.

**Figure 3:**
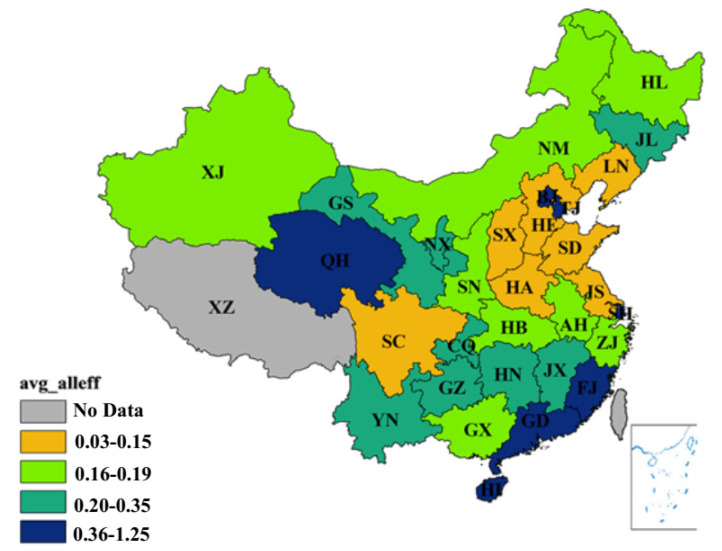
Average comprehensive efficiency from 1996 to 2015. (Source: Authors compiled).

From a spatial perspective, the eco-efficiency of different regions in China remained drastically different, which indicates a considerable gap between the regions of economic development and the effects of environmental protection effects. Figure 3 shows that from 1996 to 2015, the average eco-efficiency of the 30 different regions in China fell into four sections: 0.03–0.15, 0.16–0.19, 0.20–0.35 and 0.36–1.25. The average comprehensive eco-efficiency of Tibet remained missing. Thus, the specific categorizations are as follows. (1) The average eco-efficiency of Hainan, Guangdong, Fujian, Beijing, Tianjin, Qinghai and Shanghai fell between 0.36–1.25, which are considerably high. Among them, the eco-efficiency of Beijing continued to rise within the time span of this study. It also showed a linear increase from 2002 to 2003. (2) The average eco-efficiency of Yunnan, Guizhou, Chongqing, Hunan, Jiangxi, Ningxia, Gansu and Jilin were between 0.20–0.35 and were yet to be improved. (3) In Guangxi, Zhejiang, Anhui, Hubei, Xinjiang, Inner Mongolia and Heilongjiang, the average eco-efficiency lay within the scope of 0.16–0.19. (4). The eco-efficiency of Hebei, Shandong and Henan was in the lowest group. Hebei which is adjacent to Beijing and Tianjin has taken over a substantial number of heavy-polluting enterprises reallocated from the two regions. The less developed areas plan to transform their advantages of resource endowment into economic growth. However, this resource-driving development pattern adds the pressure on the ecological environment [49]. The average eco-efficiency of Shandong was low because of its economy’s over-reliance on the coal industry and heavy chemical industry. Moreover, the capacity of its economic development was not compatible with the environmental conditions. In Henan province, several input consumptions and emissions rank among the top in China, including capital input, energy consumption, the amounts of carbon emission and SO_2_ emission, which undermined the improvements of eco-efficiency.

### 3.2. Eco-Efficiency Analysis of China’s Regional Production Process

**Figure 4:**
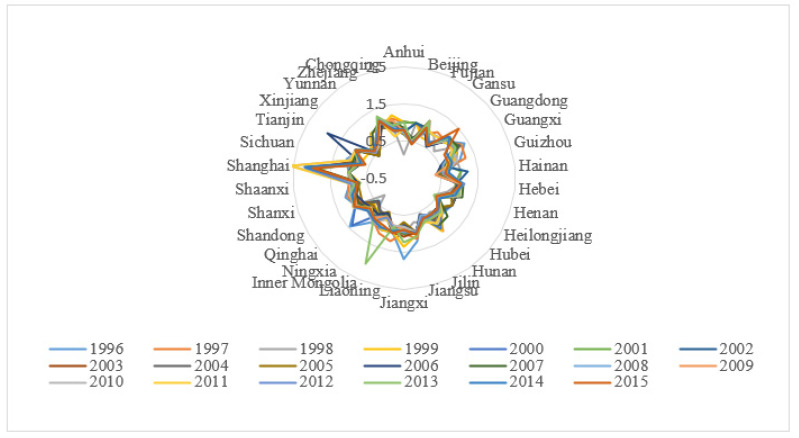
Environmental production efficiency of different provincial administrative regions in China. (Source: Authors compiled).

**Figure 5:**
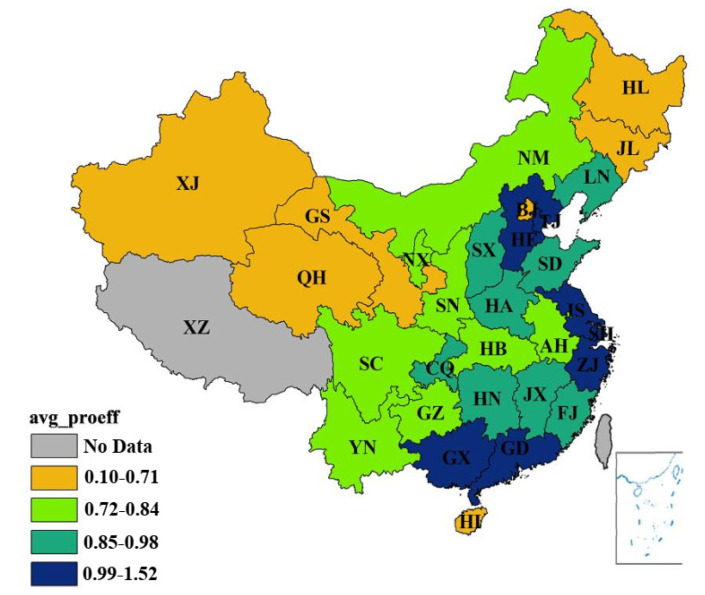
The average production efficiency from 1996 to 2015. (Source: Authors compiled).

Appendix ATable A1 illustrates the production efficiency of each province in 1996–2015. The annual changes are depicted in Figure 4. In addition, the average production efficiencies of each province are shown in Figure 5.

From a chronological perspective (Figure 4), different regions in China exhibited diverse changes in various periods between 1996 and 2015. These changes are characterized by gradual decrease, slow increase, drastic fluctuation or steadiness. In general, the environmental production efficiency of most regions in China was comparatively low. To be specific, from 1996 to 2015, the production efficiency of Guangdong and Zhejiang slowly increased. Meanwhile, the efficiency of Beijing, Guangxi, Guizhou, Heilongjiang and Huanan demonstrated a gradual decline. In addition, the production efficiency of Shanghai drastically fluctuated, and the environmental efficiency of other provincial administrative regions remained generally steady. China’s eco-efficiency has not improved simultaneously with the economic growth. In the future, China should focus not only on areas such as Beijing, Guangxi, Guizhou, Heilongjiang and Hunan, where environmental production efficiency has declined, but also on the reasons for the decline. Moreover, we should explore the experiences of areas with rising environmental productivity in Guangdong and Zhejiang, formulate corresponding policies and promote them throughout the country.

From a spatial perspective, the average production efficiency of 30 regions (municipalities) in China fell under four sections from 1996 to 2015: 0.10–0.71, 0.72–0.84, 0.85–0.98 and 0.99–1.52. An apparent gradual increase was observed from the western areas to the southeast coastal areas. However, some regions in central China, such as Heilongjiang, Jilin and some portions in the eastern part, such as Beijing and Hainan, possessed relatively low average production efficiency at 0.10–0.71. By comparison, some developed eastern provincial administrative regions, such as Jiangsu, Zhejiang and Tianjin, advanced their production technology and had better industrial structure. They were also endowed with a particularly beneficial political or economic status. Thus, these provincial administrative regions performed well in terms of production efficiency. Provincial administrative regions with low production efficiency were divided into two types. One type referred to underdeveloped regions of western China, such as some industrial-outdated regions like Guizhou, Yunnan and Xinjiang. The level of industrial development in these regions is low, and the capacity of industrial driving characteristics is limited, resulting in low production efficiency [50]. The other category was represented by Hebei and other special areas. Given their unique geographical location, substantial resources needed to be provided for the two municipalities of Beijing and Tianjin. Thus, the efficiency of these regions was lower than that of the others.

### 3.3. Analysis of the Efficiency of China’s Provincial Environmental Treatment

**Figure 6:**
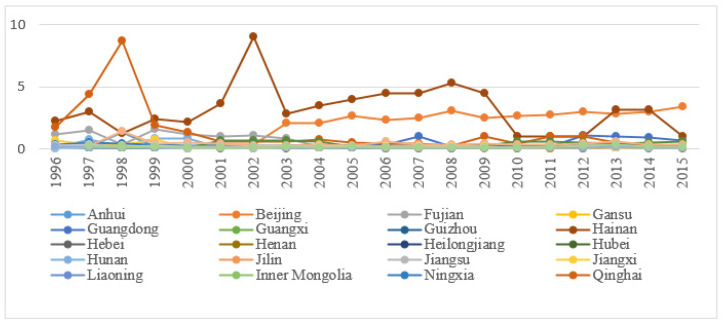
The provincial environmental treatment efficiency of China. (Source: Authors compiled).

**Figure 7:**
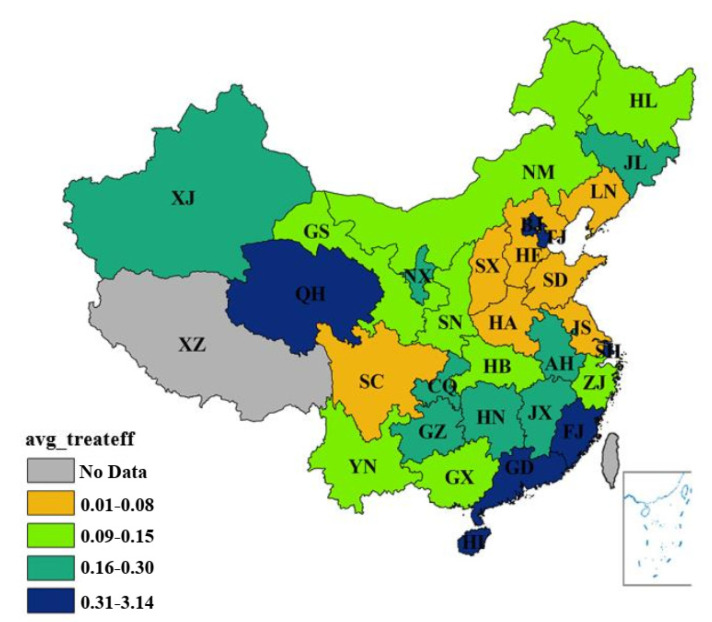
The average environmental treatment efficiency between 1996 and 2015. (Source: Authors compiled).

**Figure 8:**
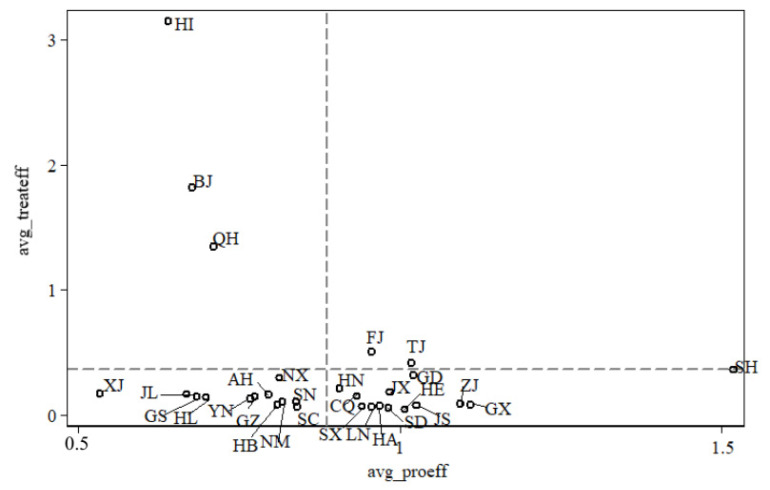
Two-dimensional graph of China’s provincial eco-efficiency distribution. (Source: Authors compiled). Note: The horizontal axis represents the production efficiency on average, and the vertical axis represents environmental treatment efficiency on average.

Appendix ATable A2 displays the efficiency of the environmental treatment stage of different provincial administrative regions from 1996 to 2015. The estimation results are shown in Figure 6, Figure 7 and Figure 8.

From a chronological perspective (see Figure 6), the environmental treatment efficiency in most provinces was low from 1996 to 2015. In addition, the amplitudes were minor. Between 1996 and 2015, the environmental treatment efficiency of Qinghai dramatically increased in 2008, thus placing the city at the highest ranking that year. However, the environmental treatment efficiency of Qinghai continuously decreased in the following years. In 2002, the efficiency of Hainan peaked and held the top spot. Overall, the environmental treatment efficiency of most regions was low. Meanwhile, the efficiency steadily increased in regions with prominent fluctuations, including Beijing. The efficiency of Hainan and Qing Hai, which had drastic fluctuations, first rose and then declined.

From a spatial perspective, the environmental treatment efficiency of 30 provincial-level regions in China increased from the north to the south between 1996 and 2015. However, the environmental treatment of Beijing, Tianjin and Qinghai ranked among the top areas. Beijing and Tianjin represented highly urbanized areas, while Qinghai represented less economically developed areas whose economy depended on tourism. Particularly, in order to promote ecological protection, Beijing and Tianjin have accelerated the upgrading and restructuring of energy consumption and industrial structure, improved energy utilization efficiency and reduced the proportion of traditional fossil-based energy such as coal and oil [48]. Economic development was bound to sacrifice the ecosystem to some extent. Nevertheless, strong economic capacity could solve ecological problems in the process of development, and the ecological recompense mechanism was mature. Although the environmental efficiency of Jiangsu and Zhejiang was relatively low, its economic efficiency was at a high level. Therefore, the eco-efficiency of the regions remained at the forefront of the country. In contrast, the environmental treatment efficiency of Heilongjiang, Jilin and Liaoning was considerably low because of the limitations brought by technology and industrial structure. Most of the time, environmental treatment was lower than 0.01.

In Figure 8, the two-dimensional graph of China’s provincial eco-efficiency distribution compares the production efficiency with the environmental treatment efficiency during the two stages of economic activities. Some important findings were derived. (1) Beijing, Qinghai and Hainan belonged to the upper-left quadrant, which was characterized by a high level of environmental treatment efficiency with a low level of production efficiency. (2) Fujian and Tianjin were both located in the upper-right quadrant, which indicated that the values of production efficiency and the values of environmental treatment of both provincial administrative regions were higher than the average. (3) The production efficiency and environmental treatment were low in provincial administrative regions with economies that were lagging behind, including Gansu, Guizhou, Ningxia, Hebei, Jilin, Sichuan, Xinjiang, Yunnan, Hubei, Shanxi and Heilongjiang. Moreover, these provincial administrative regions all fell within the lower-left quadrant. (4) As for economically well-developed regions, such as Shanghai, Zhejiang, Jiangsu, Hunan, Guangdong, Guangxi, Chongqing, Jiangxi, Liaoning, Shandong, Henan and Shanxi, the efficiency of the production stage evidently outweighed the average efficiency. In addition, that of the environmental treatment stage was prominently lower than the average value. The comprehensive efficiency of these provincial administrative regions ranked at the top in the country, proving that economic development could indeed facilitate environmental improvement.

## 4. Analysis of the Driving Factors of China’s Eco-Efficiency

### 4.1. Selection of Indexes

According to the DPSIR model, five factors, including driving forces, pressure, state, impact and response are used in the analysis of the driving factors of eco-efficiency. With reference to existing research, Table 3 lists the relevant influential variables of first-class and second-class eco-efficiency.

This section investigates the driving factors of the eco-efficiency of two different stages, namely, the production and environmental treatment stages.

### 4.2. The Construction of the Estimation Model

Correlation analysis and partial correlation analysis were originally intended to eliminate indicators that are not significantly related to the relevant variables. The first model of structural equations is constructed with an index on the left. After obtaining the external load factors of the main and secondary indexes, the sub-index (include the index less than 0) is excluded from the DPSIR model, which is consistent with the PLS-SEM. The next step is to evaluate the validity of distinguishing validity. The AVE square root should be greater than the coefficient of the correlation between its exponent and other indexes. In this way, the selected main indicator can be regarded as a different indicator with good effectiveness. The relevant data are shown in Table 4, Table 5 and Table 6.

Table 4 illustrates the ultimate outcomes of the indexes, which influence the eco-efficiency of various stages. At the same time, the estimated structural pathways of various stages in the DPSIR model are calculated and examined with the PLS method. To determine the reliability and validity the DPSIR model, the convergence reliability (CR) needs to be higher than the minimal requirement of −0.7. Moreover, the AVE must surpass 0.5 and be larger than the relevant coefficient of primary indexes simultaneously. As shown in Table 4, the CR and AVE meet the minimal requirement, which means that the model is reliable and valid. 

### 4.3. Analysis of the Driving Factors of the Production Stage

**Figure 9:**
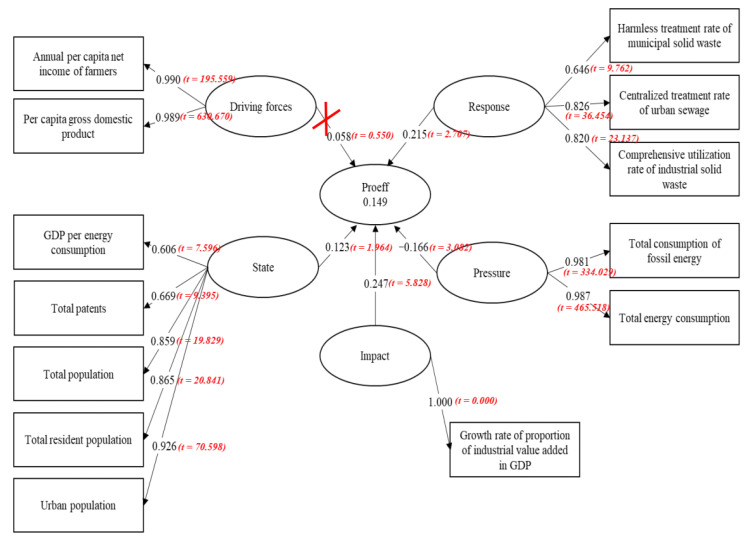
Influencing factors of efficiency in production stage based on DPSIR and PLS-SEM. (Source: Authors compiled).

Figure 9 shows the empirical findings of the impact (the influence coefficient is 0.247), response (the influence coefficient is 0.215), state (the influence coefficient is 0.123) and pressure (the influence coefficient is −0.166). All four factors exert significant impacts on the eco-efficiency of the production stage. The specific explanations are as follows.

(1) The influence coefficient between impact and production efficiency is 0.247, which indicates that the factor of impact has prominent positive influence on the environmental production efficiency. Its influence is the largest among the indexes, which is represented by the fact that a high proportion of industrial value added in the GDP entails increased environmental production efficiency. On the one hand, the expansion of the industrial value-added proportion of GDP demonstrates the enhanced capability of industrial production. On the other hand, it indicates progressing industrial technology innovation. Improvement in industrial technology innovation also decreases the overall energy consumption and pollution brought by industrial products. To some extent, it demonstrates the improvement of the environmental production efficiency.
(2) The response system influences the production efficiency to a certain degree. The influence coefficient is 0.215, which means that a collective sewage treatment by an industrial solid waste treatment plant and its comprehensive utilization increases the production efficiency as the province puts more effort into the decontamination of urban refuse. Li and Liu [74] found that technological innovation is the principal force for promoting green and inclusive all-factor productivity. They believe that enhancing the production efficiency should be the key to numerous challenges, such as sustaining development, eradicating poverty, saving natural resources and protecting the environment.
(3) The pathway coefficient of state and production efficiency is 0.123, thus reflecting a positive influence on the production efficiency exerted by the value of the state. However, this influence is the most trivial. The production efficiency would improve correspondingly with the increase of the per capita GDP’s consumption of energy due to the number of patents being passed, the urban population, the total number of permanent population and the total population. In fact, the population, which includes the urban population and the permanent population, is a factor closely related to the environment. The increase of these parameters leads to substantial labor force provision. In addition, the labor force could significantly enhance the production efficiency. The growing number of patents indicates higher innovative ability, which also improves the production efficiency.
(4) Pressure is the only index that shows an evident negative influence on the production eco-efficiency. The pathway coefficient is −0.166, which indicates that enlarging the overall consumption of energy and fossil fuels brings about low production eco-efficiency. Therefore, a larger consumption of energy and fossil fuel lowers the energy-utilizing efficiency in China. Fossil fuels not only elicit tremendous pollution but also characterize China’s industrial structure, where the secondary industry remains the largest. This circumstance hinders the country’s adjustment of the industrial structure and the enhancement of the innovative ability. All these factors contribute to low production eco-efficiency.

### 4.4. Analysis of the Driving Factors of the Treatment Stage

**Figure 10:**
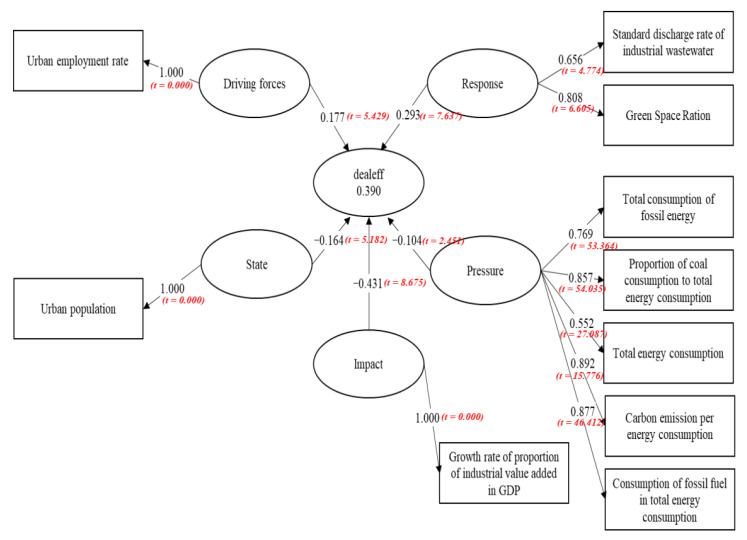
Influencing factors of efficiency in the treatment stage based on DPSIR and PLS-SEM. (Source: Authors compiled).

Figure 10 demonstrates that driving forces (the influence coefficient is 0.177), response (the influence coefficient is 0.293), impact (the influence coefficient is −0.431), pressure (the influence coefficient is −0.104) and state (the influence coefficient is −0.164) all have a significant impacts on the eco-efficiency of the management stage. To be specific:

(1) The pathway coefficient of the driving factors and the environmental treatment efficiency is 0.177. This value shows that a higher urban employment rate increases the environmental treatment efficiency correspondingly. Thus, the population and the environment are always closely connected. When urbanization continues to expand, the labor force also increases. As a result, the urban employment rate and the city’s development rise. This cycle is beneficial to the city. With the increase in the employment rate and the development of the city, the labor force in the area where environmental management is implemented also steadily increases along with the improved population quality and efficiency in environmental treatment. Therefore, regions with poor human resources bases need to increase investment in human resources development and introduce and cultivate high-tech talents, in order to promote the development of high-tech industries [48].
(2) The response system exerts a relatively large impact on the environmental treatment efficiency, which is represented by an influence coefficient of 0.293. This value indicates a higher afforestation rate. In addition, the control rate of industrial sewage discharge stimulates the environmental management rate. Afforestation is one of the crucial methods of environmental management. Generally, the high afforestation rate of a city entails better environmental treatment efficiency. Currently, water pollution is one major difficulty faced by urban environmental treatment, as the pollution caused by industrial wastewater is rather severe. Thus, when the control rate of industrial sewage discharge is higher, the city’s environmental treatment efficiency also increases.
(3) The influence coefficient of the influencing factors and the environmental treatment efficiency is −0.431, which mainly reflects an inversion between the expanding industrial proportion in the GDP value added and the environmental treatment efficiency. The most important index is the industrial proportion in the GDP value added, which signifies that industrial pollution exerts a considerable impact on the environmental treatment efficiency. Given that the ratio of the secondary industry in China is still high, the improvement of the environmental treatment efficiency is hindered, which indicates that the industrial structure of our country needs to be adjusted.
(4) Moreover, the pressure system has an evident negative impact on environmental treatment efficiency. The pathway coefficient between the two factors is −0.104, as shown by the result of the model. Carbon emission and energy consumption is an important indicator of environmental treatment efficiency. The major contributors are the consumption of fossil fuel. The high ratio of fossil fuel and coal consumption lowers the environmental treatment efficiency. The overall energy consumption, particularly the overall consumption of fossil fuel itself, greatly pollutes the environment, which is at odds with environmental treatment. Thus, a greater overall energy consumption and fossil fuel consumption increases the severity of the pollution. Moreover, the increase in consumption decreases the environmental treatment efficiency.
(5) The pathway coefficient of the state and the environmental treatment efficiency is −0.164, which means that state indeed influences environmental treatment efficiency negatively. With an increasingly urban population, environmental treatment efficiency decreases. China is still a developing country at its current stage. The concept of energy conservation and environmental protection is insufficient, which will lead to the decline of ecological efficiency [75]. From the perspective of the pathway coefficient, the influence remains comparatively low even though the index of the urban population evidently negatively affects the environmental treatment efficiency.

### 4.5. Analysis of the Driving Factors of Overall Efficiency

**Figure 11:**
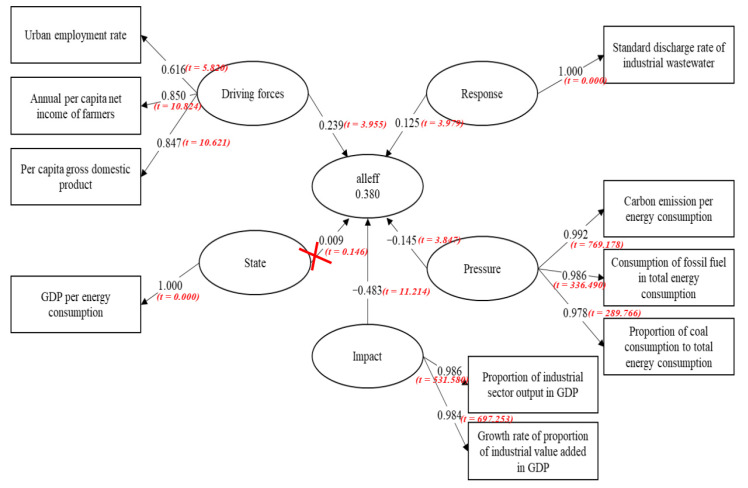
Influencing factors of efficiency in the overall stage based on DPSIR and PLS-SEM. (Source: Authors compiled).

Figure 11 demonstrate that driving forces (the influence coefficient is 0.239), response (the influence coefficient is 0.125), impact (the influence coefficient is −0.483) and pressure (the influence coefficient is −0.145) all remarkably affect the overall eco-efficiency. To be specific:

(1) The pathway coefficient of the driving forces and the overall eco-efficiency is 0.239, which means that the GDP per capita, the farmers’ net income per capita and the urban rate of employment share a positive relationship with the overall eco-efficiency. Several factors influence regional eco-efficiency. One of the widely acknowledged aspects is economic status. Moreover, the GDP per capita, the farmers’ net income per capita and the increase of the urban rate of employment can all enhance the overall eco-efficiency. Economic development has advanced science and technology, thus facilitating the improvement of the overall eco-efficiency.

(2) The response system also exerts a certain influence on the overall eco-efficiency. The influence coefficient is 0.125, which means that the control rate of the industrial sewage treatment has not reached the standard domestically. Moreover, the response system does not increase the overall eco-efficiency. Technological strength is another important demonstration of a country or region’s comprehensive strength. It is also key to economic development and environmental quality. Progress in pollution control technology can cultivate a better environment, while the development of cleaning and decontamination technology can enhance the utilizing and recycling rates of resources and reduce the pollution discharge per unit. In addition, the technology externality and the effect of technology spillover among enterprises in different regions can greatly improve the efficiency of clean technology research and development (R&D), which promote the reduction of regional pollution [76]. Although the response system influences the overall eco-efficiency to some extent, the influence coefficient is relatively minor compared with other factors.

(3) The influence coefficient between the impact and the overall eco-efficiency is −0.483. By comparison, the value of the impact has the strongest negative influence on the overall eco-efficiency. The empirical results show that the industrial proportion in the GDP value added is negatively correlated with eco-efficiency. With regard to the relationship with industrial structural changes and environmental pollution, one consensus is that when a country’s economy turns from an agriculture-based economy to an industrialized one, the environmental pollution is exacerbated. With accelerating industrialization, more resources are exploited and used, and the environment deteriorates with the increasing waste discharged. Given that waste discharge is mainly derived from industrial production, a higher ratio of industrial value added into the GDP entails a lower efficiency of resource consumption, environmental pollution and overall environment.

(4) The pressure system also exerts a prominent negative impact towards the overall eco-efficiency. The pathway coefficient between the two is −0.145, which means that this factor has the least effect on the overall eco-efficiency. In addition, the carbon emission per energy consumption unit is directly related to the environmental treatment efficiency. The major factors are the consumption of fossil fuel and coal. A higher proportion of fossil fuel and coal consumption indicates lower overall eco-efficiency. In recent years, we have actively promoted industrial transformation and upgrading. Moreover, the industrial capacity of energy-driven enterprises and heavy pollutant emission enterprises has continuously reduced. Instead, environmentally friendly industries, including the service industry, have taken over. The optimization of the industrial structure enhances eco-efficiency, as confirmed by several experts.

## 5. Conclusions

Eco-efficiency is the marginal impact of the output added value on the environmentally improved eco-efficiency. This study explores the overall production and environmental eco-efficiency of 30 provincial regions in China using the two-stage DEA model. This study finds that (1) the eco-efficiency of most Chinese regions is low, and there are regional differences evident. 2) The factors of pressure, state, impact and response exert significant effects on the eco-efficiency of the production stage. Meanwhile, all DPSIR factors have strong impacts on the eco-efficiency of the treatment stage. (3) By comparing the production efficiency with the environmental efficiency, we find that the eco-efficiency of the production stage evidently outweighs the average efficiency in economically well-developed regions.

Specifically, the main findings are elaborated as follows.

(1) The overall eco-efficiency of various provincial administrative regions manifests greatly different trends from 1996 to 2015. In most regions, the eco-efficiency was below 1, indicating that they were less efficient. From a spatial perspective, eco-efficiency varied greatly between 30 provincial administrative regions due to different levels of economic development and environmental protection. The average eco-efficiencies of 30 different regions in China fell into four sections: 0.03–0.15, 0.16–0.19, 0.20–0.35 and 0.36–1.25. The average eco-efficiencies of Hainan, Guangdong, Fujian, Beijing, Tianjin, Qinghai and Shanghai were between 0.36 and 1.25, which are considerably high. Meanwhile, the eco-efficiencies of Hebei, Shandong and Henan were in the lowest group. In addition, the trend from the western areas to the southeast coastal areas gradually increased. From a chronological perspective, the production of most regions in China was comparatively low, without much fluctuation.
(2) The two-stage DEA model divides the eco-efficiency into production efficiency and treatment efficiency. From a chronological perspective, different regions in China display diverse changes in various periods between 1996 and 2015. In general, the environmental production efficiency of most regions in China was comparatively low. The environmental treatment efficiency was also low, and the amplitudes were minor. From a spatial perspective, the average production efficiency of 30 regions (municipalities) in China fell into four sections: 0.10–0.71, 0.72–0.84, 0.85–0.98 and 0.99–1.52. An apparent trend of gradual increase was also observed from the western areas to the southeast coastal areas. Moreover, the environmental treatment efficiency of 30 provincial administrative regions in China increased from the north to the south.
(3) When comparing the production efficiency with the environmental treatment efficiency, some important findings can be derived. Beijing, Qinghai and Hainan efficiency and the environmental treatment of Fujian and Tianjin were higher than the average. These regions show low levels of production efficiency and high levels of environmental treatment efficiency. The production efficiency and environmental treatment were low in the group of provincial administrative regions where economies were lagging behind the national average. For economically well-developed regions, the efficiency of the production stage evidently outweighed the average efficiency. Meanwhile, that of the environmental treatment stage was prominently lower than the average value.
(4) Impact, response, state and pressure have remarkable influences on the eco-efficiency of the production stage, where the value of impact is the most influential among the indexes. Aside from the pressure system, other indexes all exerted positive influences on production efficiency. The driving forces, response, impact, pressure and state all significantly influenced the eco-efficiency of the management stage, where the response index was more influential. Except for driving forces and impact, other indexes negatively affected eco-efficiency. In terms of the overall eco-efficiency, the driving forces, response, impact and pressure were all important influencing factors, where impact exerted the most influence. The relationship between the driving forces and the overall eco-efficiency was positive, and the same was true with the response. Meanwhile, the influences of impact and pressure were negative.

According to the estimation results, this study reveals important findings. There are remarkable differences in ecological efficiency among different provinces in China, and most of them are in non-efficiency state, which indicates that China’s economic development in the past was at the expense of ecological protection to varying degrees, and this traditional economic development mode will not bring sustainable economic development. Ecological environment governance is an imperative with positive externality. In order to promote the transition of China’s economy to high-quality development, this paper puts forward the following policy recommendations:

Firstly, ex ante supervision and governance of ecological environment and legal deterrence are vitally important. The central and local governments should reinforce the legislations of environmental protection laws and regulations, and strengthened the punishment of environmental misconducts. Secondly, supportive institutional arrangements are needed to ensure the consistent implement of environmental protection policy. On the one hand, the government should encourage enterprises to recycle pollutants and upgrade green production processes by means of financial assistance and tax reduction. On the other hand, the government should strengthen the protection of intellectual property rights, which is conducive to encouraging enterprises to invent and deploy innovative or environmental friendly products. Thirdly, local governments should strengthen cross-boundary cooperation. The production efficiency and environmental treatment efficiency of Fujian and Tianjin are higher than the national average level. Local governments can learn from the experience of energy consumption restructure and industrial adjustment of these two regions. In addition, the central government should play a proactive role as a coordinator to promote balanced regional development and environmental protection. Industrial upgrading experienced in China’s coastal developed regions leads to the transfer of low-end industries to less developed regions and results in various environmental problems. Therefore, the central government should explore the cooperative path of environmental governance.

This study proposes several promising directions for future study to address the limitation of this study. With the weak disposability of undesirable outputs, it is a challenging issue to appropriately manage the potential trade-off between undesirable output abatement and desirable output production. A decision-maker may have their specific preference on the substitution rate between them in different circumstances, and changes in the substitution rate may largely affect the measurement result. To address this issue, a sensitivity analysis of the substitution rate is important to adequately control the trade-off. For example, the robustness of eco-efficiency estimation results is prone to selections of substitution rate. Such an analysis could derive rich managerial implications to alleviate the environmental impacts of socioeconomic activities.

## Figures and Tables

**Table 1:** Input-output index in different subsystems.

Subsystem	Input-Output Index
Production process	Number of labor force (Ten thousand people) (Input)
	Fixed asset investment (Billion Yuan) (Input)
	Energy consumption (Ten thousand tons standard coal) (Input)
	The Land used (Square kilometers) (Input)
	Water (Ten thousand tons) (Input)
	GDP (Billion Yuan) (Output)
Two-stage connection volume	Wastewater discharge (Ten thousand tons)
	Exhaust emissions (tons)
	SO_2_ emissions (tons)
Treatment process	Investment in pollution control (Ten thousand Yuan) (Input)
	Solid Waste utilization Rate (%) (Output)
	Wastewater Treatment Compliance Rate (%) (Output)
	Greening rate in built-up area (%) (Output)

Source: Authors compiled.

**Table 2:** China’s provincial comprehensive eco-efficiency.

Province	1996	1997	1998	1999	2000	2001	2002	2003	2004	2005	2006	2007	2008	2009	2010	2011	2012	2013	2014	2015
Anhui	0.15	0.20	0.19	0.23	0.23	0.18	0.10	0.12	0.24	0.20	0.21	0.23	0.14	0.19	0.17	0.14	0.14	0.12	0.09	0.07
Beijing	0.17	0.19	0.26	0.28	0.25	0.38	0.36	1.10	1.13	1.14	1.14	1.16	1.18	1.16	1.17	1.17	1.18	1.18	1.19	1.20
Fujian	1.05	1.07	0.38	1.05	1.03	1.01	1.00	1.00	0.24	0.19	0.21	0.25	0.21	0.30	0.25	0.22	0.28	0.30	0.19	0.16
Gansu	0.10	0.13	0.10	0.12	0.08	0.12	0.07	0.12	0.31	0.26	0.22	0.27	0.25	0.29	0.27	0.47	0.30	0.23	0.18	0.19
Guangdong	0.10	0.16	0.16	0.09	0.11	0.10	0.09	0.09	0.16	0.24	0.49	1.05	0.23	0.24	0.12	0.24	1.05	1.02	1.01	1.02
Guangxi	0.17	0.28	0.21	0.20	0.14	0.21	0.20	0.20	0.29	0.22	0.23	0.25	0.16	0.19	0.17	0.16	0.20	0.16	0.11	0.09
Guizhou	0.20	0.21	0.11	0.17	0.15	0.16	0.09	0.12	0.32	0.33	0.29	0.39	0.34	0.50	0.48	0.30	0.35	0.18	0.11	0.11
Hainan	1.17	1.13	1.15	1.36	1.05	1.91	1.92	1.29	1.32	1.30	1.29	1.31	1.54	1.27	0.78	0.76	0.78	1.40	1.40	0.77
Hebei	0.10	0.11	0.08	0.10	0.07	0.07	0.05	0.07	0.11	0.09	0.09	0.11	0.09	0.12	0.09	0.04	0.06	0.12	0.12	0.12
Henan	0.07	0.10	0.09	0.15	0.12	0.12	0.08	0.10	0.20	0.13	0.12	0.16	0.15	0.20	0.23	0.26	0.25	0.17	0.12	0.11
Heilongjiang	0.10	0.13	0.10	0.18	0.15	0.10	0.13	0.19	0.23	0.35	0.23	0.28	0.16	0.20	0.19	0.21	0.23	0.14	0.15	0.15
Hubei	0.13	0.18	0.12	0.16	0.11	0.09	0.07	0.09	0.21	0.17	0.17	0.28	0.18	0.17	0.20	0.15	0.15	0.16	0.10	0.10
Hunan	0.16	1.00	0.20	1.00	1.00	0.12	0.09	0.14	0.33	0.24	0.20	0.27	0.18	0.17	0.27	0.28	0.21	0.16	0.11	0.07
Jilin	0.10	0.17	0.15	0.20	0.11	0.13	0.10	0.17	0.24	0.23	0.22	0.29	0.22	0.29	0.21	0.33	0.37	0.35	0.29	0.23
Jiangsu	0.12	0.14	0.11	0.11	0.16	0.08	0.07	0.07	0.10	0.14	0.09	0.18	0.13	0.17	0.21	0.14	0.20	0.16	0.07	0.07
Jiangxi	1.03	0.46	0.42	1.02	0.17	0.31	0.10	0.27	0.21	0.18	0.21	0.28	0.28	0.27	0.17	0.20	0.16	0.18	0.13	0.12
Liaoning	0.07	0.62	0.10	0.12	0.06	0.08	0.08	0.08	0.10	0.09	0.08	0.12	0.09	0.11	0.13	0.07	0.08	0.11	0.10	0.08
Inner Mongolia	0.15	0.20	0.11	0.21	0.09	0.14	0.08	0.12	0.14	0.11	0.09	0.16	0.11	0.14	0.12	0.10	0.10	1.00	0.13	0.09
Ningxia	0.38	0.56	0.48	0.26	0.24	0.17	0.17	0.22	0.27	0.33	0.28	0.32	0.32	0.42	0.42	0.43	0.54	0.44	0.40	0.29
Qinghai	1.44	1.46	1.28	1.03	1.31	0.32	0.27	0.48	0.43	0.48	0.50	0.50	0.34	0.82	0.52	0.68	0.75	0.48	0.45	0.33
Shandong	0.13	0.12	0.14	0.18	0.12	0.11	0.05	0.06	0.09	0.07	0.07	0.08	0.07	0.09	0.08	0.08	0.10	0.10	0.08	0.07
Shanxi	0.13	0.15	0.07	0.11	0.06	0.10	0.08	0.11	0.20	0.18	0.15	0.16	0.14	0.16	0.15	0.10	0.12	0.14	0.12	0.10
Shaanxi	0.17	0.22	0.11	0.11	0.07	0.11	0.08	0.09	0.17	0.17	0.21	0.23	0.18	0.20	0.16	0.32	0.29	0.23	0.15	0.14
Shanghai	0.13	0.16	0.16	0.16	0.15	1.14	1.12	1.14	1.15	0.23	0.29	0.24	0.25	0.32	1.27	1.26	1.07	0.54	1.06	1.06
Sichuan	0.09	0.12	0.08	0.11	0.07	0.08	0.05	0.09	0.12	0.12	0.14	0.15	0.15	0.20	0.27	0.20	0.19	0.12	0.08	0.09
Tianjin	0.27	0.37	1.09	0.30	0.49	0.45	0.30	0.29	0.44	0.33	1.08	0.45	0.42	0.47	0.43	0.42	0.46	0.65	0.39	0.45
Xinjiang	0.21	0.30	0.15	0.25	0.18	0.11	0.11	0.14	0.18	0.21	0.27	0.30	0.22	0.21	0.22	0.24	0.18	0.15	0.11	0.11
Yunnan	0.25	0.30	0.32	0.25	0.10	0.11	0.08	0.11	0.28	0.21	0.24	0.37	0.25	0.24	0.21	0.24	0.24	0.13	0.12	0.11
Zhejiang	0.26	0.24	0.22	0.13	0.14	0.14	0.17	0.09	0.12	0.12	0.12	0.14	0.09	0.16	0.12	0.21	0.20	0.26	0.13	0.11
Chongqing		0.42	0.23	0.34	0.20	0.18	0.14	0.25	0.21	0.22	0.19	0.28	0.25	0.25	0.19	0.21	0.36	0.38	0.27	0.27

Source: Authors compiled.

**Table 3:** Descriptions on eco-efficiency influential variables.

Aspects	Variables	Attrs	Reference
Driving forces	Per capita gross domestic product	+	[1,5]
	Population growth rate (gr_popu)	+	[1,5]
	Urbanization rate (D3) (urbaniza)	+	[6,7]
	Construction land growth rate	+	[10,11]
	Urban employment rate (D5) (employ)	+	[8]
	Annual per capita net income of farmers	+	[51]
Pressure	Population density (den_popu)	+	[52]
	Total energy consumption (cosum_enr)	+	[53]
	Energy consumption per unit of GDP	+	[1]
	Per capita energy consumption	+	[54,55]
	Proportion of coal consumption to total energy consumption	+	[56,57]
	Consumption of fossil fuel in total energy consumption	+	[9]
	Total consumption of fossil energy	+	[4]
	Carbon emission per energy consumption (carb_pener)	+	[8]
State	Total population (popu)	+	[1,58]
	Urban population (popu_urba)	+	[59]
	Total resident population	+	[60]
	Proportion of floating population to total population	+	[61]
	Average family size	+	[62]
	Total patents (patent)	-	[63]
	GDP per energy consumption (gdp_enr)	-	[64]
Impact	Proportion of industrial sector output in GDP	+	[65]
	Proportion of output value of secondary industry in GDP	+	[66]
	Proportion of the tertiary industry output in GDP (tertira)	-	[63,66]
	Growth rate of proportion of industrial value added in GDP	+	[67]
	Growth rate of proportion of agricultural value added in GDP	+	[67,68]
Response	Proportion of environmental protection investment in GDP (envrinvera)	-	[69]
	Green space ration	-	[12]
	Harmless treatment rate of municipal solid waste	-	[70]
	Comprehensive utilization rate of industrial solid waste	-	[71]
	Centralized treatment rate of urban sewage	-	[72]
	Standard discharge rate of industrial wastewater (tap water)	-	[73]

Source: Authors compiled. Note: + indicates the gain indicator, that is, a greater value denotes better gain; - indicates the expense indicator, that is, a higher value represents worse performance.

**Table 4:** External load factor.

Stage	Measurable Variables	First-Class Variables				
		Driving forces	Impact	Pressure	Response	State
Production efficiency	Total consumption of fossil energy			0.981		
	Total energy consumption			0.987		
	Proportion of environmental protection investment in GDP				0.048	
	GDP per energy consumption					0.626
	Growth rate of proportion of agricultural value added in GDP		−0.855			
	Growth rate of proportion of industrial value added in GDP		0.894			
	Population growth rate	−0.669				
	Harmless treatment rate of municipal solid waste				0.6	
	Annual per capita net income of farmers	0.928				
	Total patents					0.678
	Per capita gross domestic product	0.942				
	Total population					0.834
	Proportion of floating population to total population					0.098
	Total resident population					0.843
	Urban population					0.916
	Centralized treatment rate of urban sewage				0.803	
	Comprehensive utilization rate of industrial solid waste				0.806	
Treatment efficiency	Carbon emission per energy consumption			0.904		
	Proportion of coal consumption to total energy consumption			0.877		
	Total consumption of fossil energy			0.743		
	Consumption of fossil fuel in total energy consumption			0.888		
	Total energy consumption			0.519		
	Urban employment rate	0.965				
	Energy consumption per unit of GDP			0.188		
	GDP per energy consumption					−0.373
	Growth rate of proportion of industrial value added in GDP		1			
	Green Space Ration				0.808	
	Annual per capita net income of farmers	0.386				
	Urban population					0.652
	Standard discharge rate of industrial wastewater				0.656	
Total efficiency	Carbon emission per energy consumption			0.992		
	Proportion of coal consumption to total energy consumption			0.978		
	Consumption of fossil fuel in total energy consumption			0.986		
	Urban employment rate	0.664				
	GDP per energy consumption					1
	Growth rate of proportion of industrial value added in GDP		0.984			
	Population growth rate	−0.016				
	Annual per capita net income of farmers	0.788				
	Proportion of industrial sector output in GDP		0.986			
	Per capita gross domestic product	0.775				
	Standard discharge rate of industrial wastewater				1	

Source: Authors compiled.

**Table 5:** Discriminant validity of the DPSIR model.

Stage	DPSIR Factors	Driving Forces	Impact	Pressure	Response	State
Production efficiency	Driving forces	0.989				
	Impact	0.138	1			
	Pressure	0.468	0.518	0.984		
	Response	0.756	0.304	0.581	0.769	
	State	0.421	0.338	0.7	0.668	0.795
Treatment efficiency	DPSIR factors	Driving forces	Impact	Pressure	Response	State
	Driving forces	1				
	Impact	−0.185	1			
	Pressure	−0.098	0.43	0.799		
	Response	0.024	0.181	0.066	0.736	
	State	0.052	0.387	0.234	0.351	1
Total efficiency	DPSIR factors	Driving forces	Impact	Pressure	Response	State
	Driving forces	0.779				
	Impact	−0.023	0.985			
	Pressure	−0.198	0.262	0.985		
	Response	−0.003	0.102	−0.129	1	
	State	0.746	−0.041	−0.361	0.142	1

Source: Authors compiled.

**Table 6:** Structural path estimates for the DPSIR model.

Stage	DPSIR Factors	Cronbach’s Alpha	rho_A	Convergence Reliability	The Average Variance Extracted (AVE)	Influence Coefficient
Production efficiency	Driving forces	0.978	0.98	0.989	0.979	0.058
	Impact	1	1	1	1	0.247
	Pressure	0.967	0.988	0.984	0.968	−0.166
	Response	0.666	0.708	0.811	0.591	0.215
	State	0.847	0.871	0.893	0.632	0.123
Treatment efficiency	Driving forces	1	1	1	1	0.177
	Impact	1	1	1	1	−0.431
	Pressure	0.849	0.865	0.896	0.639	−0.104
	Response	0.158	0.164	0.701	0.542	0.293
	State	1	1	1	1	−0.164
Total efficiency	Driving forces	0.668	0.636	0.819	0.607	0.239
	Impact	0.97	0.974	0.985	0.971	−0.483
	Pressure	0.985	1.02	0.99	0.971	−0.145
	Response	1	1	1	1	0.125
	State	1	1	1	1	0.009

Source: Authors compiled.

## Appendix A

**Table A1:** China’s provincial environmental production efficiency.

Provinces	1996	1997	1998	1999	2000	2001	2002	2003	2004	2005	2006	2007	2008	2009	2010	2011	2012	2013	2014	2015
Anhui	0.75	1.00	0.13	1.00	1.00	1.00	0.74	0.76	0.78	0.68	0.75	0.84	0.71	0.75	0.78	0.82	0.82	1.01	0.80	0.79
Beijing	1.00	1.00	1.00	1.00	1.00	1.00	1.00	0.58	0.61	0.49	0.56	0.53	0.44	0.53	0.50	0.49	0.46	0.48	0.46	0.41
Fujian	0.93	0.78	1.00	0.70	0.90	0.98	0.97	1.19	0.89	0.90	0.88	1.01	0.89	0.96	1.10	0.97	0.95	1.18	0.96	0.98
Gansu	0.91	1.04	0.87	0.68	0.64	0.62	0.57	0.63	0.64	0.59	0.54	0.61	0.67	0.66	0.60	0.91	0.69	0.60	0.63	0.57
Guangdong	1.00	1.00	0.58	0.92	1.00	1.00	1.00	1.00	1.00	1.00	1.00	1.14	1.00	1.00	1.00	1.00	1.04	1.08	1.16	1.50
Guangxi	1.37	1.29	1.16	1.19	1.16	1.26	1.22	1.19	1.24	1.06	1.05	1.17	1.03	1.03	1.01	1.01	1.09	1.10	0.82	0.75
Guizhou	1.07	1.24	1.00	0.90	0.69	0.66	0.58	0.53	0.55	0.56	0.54	0.71	0.60	0.87	0.99	0.73	0.80	0.80	0.88	0.76
Hainan	0.62	0.44	1.06	0.72	0.50	1.05	1.23	0.64	0.55	0.45	0.38	0.39	0.57	0.36	0.62	0.59	0.58	0.73	0.71	0.63
Hebei	0.98	1.00	1.00	0.88	0.92	0.89	0.93	1.00	1.09	0.99	1.00	1.11	1.00	1.01	1.01	1.06	1.14	1.01	1.07	1.06
Henan	1.00	1.00	0.75	1.00	1.00	1.00	1.00	1.00	0.99	0.84	0.86	1.16	0.97	0.93	1.09	1.05	0.89	1.04	0.97	0.85
Heilongjiang	1.00	1.00	0.44	0.61	1.00	0.50	1.00	1.00	1.00	1.00	0.52	0.58	0.52	0.49	0.58	0.58	0.59	0.45	0.62	0.51
Hubei	0.91	0.77	0.89	0.89	0.86	0.74	0.72	0.77	0.81	0.70	0.71	1.06	0.75	0.73	0.85	0.88	0.81	0.86	0.76	0.73
Hunan	0.87	1.28	0.65	1.26	1.18	0.90	0.90	0.86	0.99	0.76	0.71	1.09	0.73	0.75	1.05	1.00	0.84	0.83	0.73	0.73
Jilin	0.63	0.63	0.84	0.67	0.62	0.60	0.56	0.59	0.61	0.61	0.60	0.68	0.62	0.72	0.66	0.81	0.67	0.80	0.72	0.72
Jiangsu	1.24	1.17	0.72	1.09	1.00	1.03	1.03	1.00	1.00	1.00	1.00	1.00	1.00	1.00	1.00	1.00	1.00	1.12	1.04	1.05
Jiangxi	1.68	1.00	1.00	1.36	1.00	1.00	0.74	1.08	0.80	0.70	0.79	1.00	0.97	0.87	0.82	0.91	0.94	1.24	0.89	0.90
Liaoning	1.00	1.24	1.00	1.00	1.00	1.00	1.00	1.00	0.90	0.96	0.90	0.90	0.93	0.82	0.80	0.91	0.89	0.85	0.98	1.04
Inner Mongolia	1.04	1.14	0.90	1.03	0.68	0.67	0.67	0.64	0.60	0.51	0.54	0.61	0.64	0.65	0.75	0.69	0.67	2.04	0.98	0.90
Ningxia	0.98	0.93	1.00	0.72	0.76	0.65	0.61	0.60	0.61	0.71	0.72	0.82	0.83	0.84	0.97	0.89	0.93	0.90	0.92	0.86
Qinghai	1.47	0.62	0.20	0.55	1.40	0.43	0.41	0.46	0.47	0.61	0.69	0.73	0.76	0.82	0.82	0.70	0.76	0.75	0.81	0.73
Shandong	1.00	1.00	1.00	1.00	1.00	1.00	0.95	0.88	0.98	0.82	0.79	0.91	0.99	0.98	1.07	1.09	1.07	1.02	1.05	1.05
Shanxi	1.17	1.11	1.00	0.93	0.84	0.85	0.84	0.90	0.96	0.75	0.88	0.92	1.04	0.90	1.01	0.93	0.95	1.00	0.96	0.87
Shaanxi	0.99	1.01	0.95	0.71	0.78	0.70	0.72	0.77	0.84	0.75	0.76	0.84	0.90	0.87	0.89	0.96	0.93	0.86	0.77	0.77
Shanghai	1.00	1.00	1.00	1.00	1.00	1.84	1.94	1.93	2.18	1.00	1.00	1.00	1.00	1.00	2.58	2.58	2.16	1.00	2.19	1.97
Sichuan	1.14	0.90	0.91	0.75	0.87	0.80	0.81	0.99	0.79	0.71	0.73	0.96	0.78	0.82	1.08	1.00	0.66	0.77	0.71	0.61
Tianjin	0.92	1.02	0.87	0.67	1.00	1.00	1.00	1.00	1.00	1.00	1.89	1.00	1.00	1.00	1.00	1.00	1.00	1.00	1.00	1.00
Xinjiang	0.68	0.64	0.59	0.68	0.41	0.40	0.41	0.40	0.44	0.41	0.52	0.59	0.52	0.51	0.54	0.58	0.54	0.63	0.63	0.56
Yunnan	1.00	1.00	1.00	1.00	0.68	0.63	0.62	0.69	0.81	0.59	0.64	0.98	0.67	0.62	0.62	0.81	0.79	0.69	0.78	0.72
Zhejiang	1.00	1.00	1.00	1.00	1.00	1.00	1.00	1.00	1.00	1.04	1.03	1.13	1.18	1.18	1.18	1.26	1.24	1.32	1.15	1.18
Chongqing		1.14	0.87	1.21	0.98	0.93	0.89	0.90	1.04	0.85	1.04	1.05	0.93	1.06	0.85	0.65	0.77	0.94	0.84	0.79

**Table A2:** China’s provincial environmental treatment efficiency.

Provinces	1996	1997	1998	1999	2000	2001	2002	2003	2004	2005	2006	2007	2008	2009	2010	2011	2012	2013	2014	2015
Anhui	0.13	0.15	1.30	0.15	0.14	0.12	0.09	0.09	0.13	0.13	0.11	0.11	0.09	0.10	0.08	0.06	0.07	0.07	0.06	0.05
Beijing	0.14	0.15	0.23	0.25	0.18	0.34	0.36	2.10	2.06	2.62	2.31	2.48	3.07	2.50	2.69	2.70	2.95	2.82	3.00	3.38
Fujian	1.19	1.45	0.35	1.59	1.19	1.03	1.04	0.84	0.14	0.12	0.11	0.11	0.11	0.14	0.11	0.10	0.14	0.19	0.11	0.09
Gansu	0.07	0.08	0.08	0.11	0.08	0.11	0.08	0.10	0.18	0.18	0.15	0.16	0.15	0.18	0.17	0.33	0.18	0.22	0.16	0.15
Guangdong	0.06	0.11	0.20	0.06	0.07	0.07	0.06	0.06	0.09	0.15	0.36	0.97	0.13	0.14	0.09	0.13	1.06	0.96	0.88	0.69
Guangxi	0.07	0.13	0.12	0.10	0.07	0.09	0.09	0.08	0.11	0.09	0.09	0.09	0.07	0.08	0.07	0.06	0.07	0.08	0.07	0.06
Guizhou	0.11	0.11	0.07	0.10	0.10	0.12	0.09	0.11	0.19	0.23	0.18	0.20	0.21	0.30	0.29	0.14	0.21	0.10	0.06	0.06
Hainan	2.22	2.95	1.27	2.39	2.19	3.64	9.02	2.81	3.45	3.96	4.46	4.45	5.28	4.49	1.00	1.00	1.00	3.13	3.18	1.00
Hebei	0.05	0.07	0.05	0.06	0.04	0.04	0.03	0.04	0.04	0.05	0.04	0.04	0.04	0.05	0.04	0.02	0.02	0.06	0.06	0.05
Henan	0.05	0.07	0.09	0.09	0.07	0.07	0.05	0.06	0.09	0.06	0.06	0.06	0.06	0.09	0.09	0.11	0.13	0.08	0.06	0.05
Heilongjiang	0.07	0.08	0.19	0.19	0.10	0.14	0.11	0.13	0.14	0.23	0.21	0.19	0.12	0.15	0.11	0.12	0.14	0.18	0.12	0.12
Hubei	0.08	0.12	0.09	0.08	0.07	0.07	0.06	0.06	0.10	0.10	0.09	0.11	0.10	0.10	0.10	0.06	0.07	0.10	0.07	0.06
Hunan	0.11	0.78	0.19	0.79	0.85	0.09	0.06	0.08	0.15	0.13	0.11	0.11	0.10	0.09	0.11	0.12	0.11	0.10	0.07	0.05
Jilin	0.12	0.16	0.11	0.20	0.12	0.15	0.12	0.15	0.17	0.17	0.15	0.18	0.15	0.17	0.12	0.18	0.29	0.25	0.19	0.14
Jiangsu	0.05	0.08	0.14	0.06	0.09	0.05	0.04	0.05	0.06	0.09	0.05	0.11	0.08	0.10	0.12	0.07	0.11	0.09	0.04	0.04
Jiangxi	0.63	0.30	0.31	0.77	0.10	0.20	0.07	0.12	0.11	0.12	0.11	0.12	0.12	0.12	0.09	0.09	0.08	0.09	0.08	0.06
Liaoning	0.04	0.32	0.07	0.06	0.04	0.05	0.05	0.06	0.06	0.05	0.04	0.05	0.04	0.06	0.06	0.03	0.04	0.07	0.05	0.04
Inner Mongolia	0.09	0.10	0.09	0.11	0.07	0.12	0.07	0.08	0.09	0.09	0.07	0.09	0.07	0.09	0.06	0.07	0.06	0.49	0.08	0.06
Ningxia	0.34	0.47	0.39	0.31	0.21	0.22	0.23	0.23	0.29	0.29	0.23	0.24	0.25	0.34	0.25	0.29	0.40	0.39	0.33	0.22
Qinghai	1.76	4.41	8.68	1.93	1.35	0.58	0.56	0.62	0.74	0.49	0.45	0.40	0.29	1.00	0.46	1.00	1.00	0.50	0.37	0.28
Shandong	0.10	0.09	0.11	0.12	0.07	0.08	0.04	0.05	0.05	0.05	0.04	0.04	0.04	0.04	0.04	0.03	0.04	0.06	0.04	0.04
Shanxi	0.07	0.08	0.05	0.06	0.04	0.07	0.06	0.07	0.10	0.10	0.07	0.07	0.07	0.08	0.07	0.04	0.05	0.08	0.06	0.06
Shaanxi	0.09	0.12	0.08	0.09	0.05	0.09	0.08	0.07	0.09	0.10	0.12	0.12	0.09	0.11	0.08	0.16	0.15	0.16	0.12	0.09
Shanghai	0.08	0.11	0.12	0.10	0.10	0.69	0.64	0.67	0.60	0.16	0.20	0.14	0.17	0.22	0.59	0.59	0.53	0.44	0.51	0.57
Sichuan	0.04	0.07	0.06	0.07	0.04	0.05	0.04	0.05	0.06	0.08	0.07	0.06	0.07	0.09	0.10	0.08	0.13	0.08	0.06	0.06
Tianjin	0.22	0.26	1.37	0.38	0.51	0.39	0.29	0.26	0.34	0.27	0.62	0.31	0.31	0.42	0.33	0.37	0.39	0.58	0.37	0.36
Xinjiang	0.21	0.31	0.21	0.22	0.19	0.18	0.17	0.17	0.16	0.20	0.18	0.17	0.15	0.16	0.13	0.15	0.13	0.13	0.10	0.10
Yunnan	0.19	0.20	0.27	0.17	0.09	0.11	0.09	0.09	0.14	0.14	0.14	0.15	0.14	0.14	0.12	0.10	0.11	0.09	0.06	0.06
Zhejiang	0.16	0.17	0.17	0.09	0.09	0.08	0.10	0.06	0.07	0.07	0.06	0.06	0.05	0.07	0.05	0.09	0.08	0.13	0.07	0.06
Chongqing		0.23	0.18	0.15	0.11	0.12	0.10	0.13	0.10	0.14	0.09	0.12	0.13	0.12	0.10	0.16	0.23	0.29	0.20	0.18
